# *WDR45* Mutation Impairs the Autophagic Degradation of Transferrin Receptor and Promotes Ferroptosis

**DOI:** 10.3389/fmolb.2021.645831

**Published:** 2021-05-03

**Authors:** Qiuhong Xiong, Xin Li, Wenjing Li, Guangxin Chen, Han Xiao, Ping Li, Changxin Wu

**Affiliations:** Institutes of Biomedical Sciences, Key Laboratory of Chemical Biology and Molecular Engineering of Ministry of Education, Shanxi University, Taiyuan, China

**Keywords:** WDR45, autophagy, TfRC, iron accumulation, ferroptosis, BPAN

## Abstract

WDR45 is an autophagy-related protein that involves in the formation of autophagosome. Mutations in *WDR45* lead to the impairment of autophagy which is associated with the human β-propeller protein-associated neurodegeneration (BPAN). However, the relationship between autophagy and brain iron accumulation in patients with BPAN remains unclear. Here, we demonstrated that transferrin receptor (TfRC) which is critical for the iron import of cells was degraded *via* autophagy. TfRC was accumulated after the inhibition of autophagy by treatment with autophagic inhibitor chloroquine or knockdown of *ATG2A*. The intracellular iron content was increased in cells overexpressing TfRC or mutant WDR45, however, ferritin H (FTH) chain was decreased. Increased TfRC and simultaneously decreased FTH consequently resulted in an elevated level of ferrous iron (Fe^2+^) which further promoted cell ferroptosis, demonstrated by the increased lipid peroxidation and reactive oxygen species (ROS) and the decreased glutathione peroxidase 4 (GPX4) and cell viability. Taken together, these findings provide a piece of important evidence that WDR45 deficiency impairs autophagic degradation of TfRC, therefore leading to iron accumulation, and the elevated iron promotes ferroptosis which may contribute to the progression of BPAN.

## Introduction

Autophagy is an evolutionarily conserved lysosomal degradation pathway that plays an important role in maintaining cell homeostasis (Klionsky and Emr, 2000). The first autophagy-related (*ATG*) gene was identified by the 2016 Nobel laureate Yoshinori Ohsumi in yeast in 1993. Now the number of *ATG* genes has increased to about 40 (Staiano and Zappa, 2019). Among them, the autophagy gene *WDR45* (also known as WIPI4) was critical for the formation of the autophagosome (Bakula et al., 2017, 2018). Mutations in *WDR45* were found to be associated with a human neurodegenerative disorder, namely, β-propeller protein-associated neurodegeneration (BPAN, OMIM 300894), which is an X-linked neurodegenerative disorder characterized by a childhood onset of intellectual impairment followed by a second period of deterioration in adulthood (Araujo et al., 2017). Brain MRI of BPAN patient presents an iron accumulation in the globus pallidus and substantia nigra (Haack et al., 2012). Studies have demonstrated that mutations in *WDR45* result in the impairment of autophagy which indeed results in the pathogenesis of BPAN (Saitsu et al., 2013; Wan et al., 2019). In a previous study, we identified a novel *de novo* mutation in *WDR45* (NM_00128148.3, c.1037_1038del, pGlu346GlyfsTer7) in a Chinese girl. The deletion of these two base pairs led to a frameshift, which resulted in a truncated protein. We also confirmed that the overexpression of this mutant *WDR45* in HeLa cells impaired autophagy (Xiong et al., 2019). Furthermore, WDR45 mutant fibroblasts showed reduced autophagy and elevated iron content (Seibler et al., 2018). In 2014, Mancias et al. (2014) identified that the iron storage protein, namely ferritin, was degraded *via* autophagy in an NCOA4-dependent manner and suggested that autophagy also regulates iron homeostasis. However, how WDR45 deficiency leads to iron accumulation remains unclear.

The iron deposition played a key role in the pathogenesis of neurodegenerative diseases, excessive iron in rat hippocampus induced neuronal apoptosis accompanied by a decline in learning and memory function (Huo et al., 2019), and elevated brain iron level in human increased the risk of the onset of neurodegenerative diseases (Bartzokis et al., 2004). Transferrin receptor (TfRC) is a membrane protein that is essential for most cells to import iron under physiological conditions (Gammella et al., 2017). TfRC binds one iron-laden transferrin (holoTF), then the complex of holoTF-TfRC is internalized through endocytosis mediated by clathrin, when the pH of endosome decreased to 5.5, ferric iron (Fe^3+^) is released from TF, and the iron-free TF (apoTF)-TfRC complex is recycled to the cell surface where the physiological pH allows apoTF disassociation with TfRC (Shen et al., 2018). TfRC expression is correlated with poorer outcomes in several cancers, the overexpression of TfRC provided more irons to meet the metabolic needs of the cancer cells while downregulation of TfRC has inhibited the growth of tumor (Jiang et al., 2010; Pham et al., 2014; Voth et al., 2015; Shen et al., 2018).

Transferrin receptor could be degraded through both proteasomal and lysosomal pathways (Horonchik and Wessling-Resnick, 2008; Sirohi et al., 2013, 2015). Fujita et al. (2013) identified that the ubiquitin ligase, membrane-associated RING-CH (MARCH) 8, ubiquitinates TfRC and promotes its lysosomal degradation (Tachiyama et al., 2011). However, whether *WDR45* mutation impairs autophagic degradation of TfRC and TfRC involves in iron accumulation in patients with BPAN are still unknown. In this study, we confirmed that TfRC was indeed degraded *via* autophagy, *WDR45* mutation resulted in TfRC accumulation, and ferritin H (FTH) chain reduction, therefore, led to Fe^2+^ overload which further promoted ferroptosis in HeLa cells.

## Materials and Methods

### Vector Construction

cDNAs encoding the full length of TfRC (NM_00128148.3) and of WDR45 (NM_00128148.3) were amplified by PCR and subcloned into the pEGFP-C3 or Flag-tagged lentiviral expression vectors, where all the tags are in the N-terminal of proteins. The mutant WDR45 was amplified by PCR using a specific reverse primer. shRNAs were cloned into pLKO.1 vector. The primers and shRNA sequence were listed in Table 1.

**TABLE 1:** Oligos used in this study.

Oligo name	Sequence
GFP-TfRC F	GCGGAATTCTGATGATGGATCAAGCTAGATCAGC
GFP-TfRC R	GCGGGATCCTTAAAACTCATTGTCAATGTCCCAAAC
Flag-TfRC F	GCGGAATTCATGATGGATCAAGCTAGATCAGC
Flag-TfRC R	GCGGGATCCTTAAAACTCATTGTCAATGTCCCAAAC
Flag-WDR45 F	CGCCTCGAGATGACTCAACAGCCACTTCGAG
Flag-WDR45 R	CGCGAATTCCTTAAAAGTCATCATCATCACAG
Mutant WDR45 R	CGCGAATTCTCAAGGTACACGTCGAAAGCCTCTGTTG CAGTTTCCATC
ATG2A qPCR F	TCGCCCATCTCCGTCTACCTATTC
ATG2A qPCR R	TCGCCCTCCTCTTCCCTTTCATC
shRNA_*ATG2A*	CCGGCCTGGATAACACTGACCTCTTCT CGAGAAGAGGTCAGTGTTATCCAGGTTTTTTG

### Cell Culture, Cell Line Establishment, and Compound Treatment

HeLa and HEK293T cells were maintained in Dulbecco’s modified Eagle medium (BOSTER, China; PYG0004) supplemented with 10% fetal bovine serum (Biological Industries, Israel; 04-011-1A/B) and 1% penicillin/streptomycin (Solarbio, China; P1400). The Flag-TfRC stably expressing cell line was generated by lentiviral transduction, and the *WDR45*^*WT*^/*WDR45*^*MT*^ overexpressing cell line was generated as described previously (Xiong et al., 2019). The transient transfection of cells with GFP-TfRC was conducted using Lipofectamine 2000 (Invitrogen) according to the instructions by the manufacturer. The HeLa and HEK293T cells were cultured in the presence or absence of 100 μM chloroquine (CQ) for 8 h and 10 μM CQ for 24 h, respectively.

### Western Blotting

Cell lysates were prepared and separated by sodium dodecyl sulfate (SDS) gel electrophoresis, electrotransferred to a nitrocellulose membrane (GE), blocked with 5% non-fat milk in 10 mM Tris-HCl pH 8.0, 150 mM NaCl, 0.1% Tween-20 (v/v) (TBS-T buffer) for 60 min at room temperature, and incubated with the primary antibodies. The primary antibodies used were anti-LAMP1 (Proteintech, China; 21997-1-AP), TfRC antibody (Proteintech; 10084-2-AP) was used at a 1:1,000 dilution, the GFP antibody (Abclonal, China; AE012) was used at a 1:1,000 dilution, Flag M2 antibody (Sigma, F3165) was used at a 1:3,000 dilution, FTH antibody (Abcam, ab75973) was used at a 1:1,000 dilution, glutathione peroxidase 4 (GPX4) antibody (Abcam, ab125066) was used at a 1:1,000 dilution, actin antibody (Absin, China; abs125702) were used at a 1:1,000 dilution, and glyceraldehyde-3-phosphate dehydrogenase (GAPDH) antibody (Proteintech; 60004-1-AP) was used at a dilution of 1:10,000. Secondary antibodies used were anti-mouse IgG conjugated with peroxidase (POD) (Proteintech; SA00001-1) at a 1:10,000 dilution or anti-mouse IgG, 800 (LI-COR Biosciences) at a 1:250,000 dilution, anti-rabbit IgG conjugated with POD (Proteintech; SA00001-2) at a 1:10,000 dilution or anti-mouse IgG, 800 (LI-COR Biosciences). Relative protein amounts were determined using ImageJ.

### Immunofluorescence Staining and Image Analysis

Cells were grown on sterile coverslips and fixed in 4% formaldehyde for 15 min at room temperature, permeabilized, and blocked with PTB buffer (1× PBS containing 0.1% Triton X-100 and 0.1% BSA) for 1 h (Xiong et al., 2015). The fixed cells were incubated with LAMP1 antibody (Proteintech; 21997-1-AP) at a 1:100 dilution in PTB for 1 h, and secondary antibodies were Alexa-Fluor 568 conjugated donkey anti-rabbit IgG at a 1:1,000 dilution (Invitrogen). The nuclei were stained with 4′,6-Diamidino-2-phenylindole (DAPI) (Sigma). Images of fixed cells were taken using a Zeiss LSM710 Microscope with a 63 × 1.4 DIC Plan-Apochromat oil-immersion objective.

### Quantification of Cellular Iron Content

Cellular iron content was measured using the Iron Assay Kit (Sigma, MAK025) according to the protocol as described previously (Seibler et al., 2018). The absorbance at 593 nm was recorded using a Synergy H1MD plate reader (BioTek, United States). Ferrous iron (Fe^2+^) was assessed using FerroOrange probe (DojinDo, Japan) according to the instructions by the manufacturer. Briefly, the cells were transferred to a glass bottom cell culture dish (NEST, China; 801002) and cultured overnight in a 37°C incubator equilibrated with 5% CO_2_, the supernatant was discarded, and the cells were washed with hank’s balanced salt solution (HBSS) three times, FerroOrange (1 μM) was added to the cells as HBSS solution, and cells were incubated for 30 min. Cells were observed under a Zeiss LSM710 Microscope with a 63 × 1.4 DIC Plan-Apochromat oil-immersion objective.

### Measurement of Intracellular Reactive Oxygen Species Levels

The intracellular reactive oxygen species (ROS) levels were measured using a Reactive Oxygen Species Assay Kit (Beyotime Biotechnology, China; S0033S) according to the instructions by the manufacturer. Briefly, 1 × 10^4^ cells were seeded in a 96-well plate, incubated at 37°C in a 5% CO_2_ incubator for 24 h, then incubated with DCFH-DA for 20 min at 37°C, and measured at 488 nm excitation and 525 nm emission by a Synergy H1MD plate reader (BioTek).

### MTT Assay

Cell viability was measured using an 3-(4,5-Dimethyl-2-Thiazolyl)-2,5-Diphenyl Tetrazolium Bromide (MTT) assay as described previously (Yang et al., 2018). Briefly, 3,000 cells per well were plated in a 96-well plate and incubated at 37°C in a 5% CO_2_ incubator for 24 h. Cells were changed with fresh medium, added with 20 μl MTT, and incubated for another 4 h. MTT was removed and 100 μl dimethyl sulfoxide (DMSO) was added to each well. The absorbance at 570 nm was recorded using a Synergy H1MD plate reader (BioTek).

### Malondialdehyde Content Assay

Malondialdehyde (MDA) levels in the cells were measured using a commercial kit following the instructions by the manufacturer (Solarbio, China; BC0025). Briefly, 5 × 10^6^ cells were harvested in 1 ml lysis buffer and sonicated for 30 times (amplitude 20%, pulse on 3 s, and pulse off 10 s). The cell suspension was centrifuged at 8,000 *g* at 4°C for 10 min, and then 100 μl of the sample was added for the measurement, followed by the addition of 400 μl of the MDA test solution. After mixing and heating it in a boiling water bath for 30 min, the mixture was cooled down on the ice and centrifuged at 10,000 *g* for 10 min. The supernatant was taken, and the absorbance was measured at 450, 532, and 600 nm using a Synergy H1MD plate reader (BioTek). According to the instructions by the manufacturer, the levels of MDA were evaluated and calculated by the following formula:

$$\mathrm{MDA}\mathrm{content}\left(\mathrm{nmol}/\mathrm{mg}\mathrm{protein}\right)=5\times \left(12.9\times \left(A_{532}-A_{600}\right)-2.58\times A_{450}\right)/C_{\text{protein}}$$

### Statistical Analysis

The densitometry analysis was performed by using ImageJ. The differences were analyzed statistically using the *t*-test. The error bars indicate SD of the mean of *N* ≥ 3 independent experiments (^∗^*p* < 0.05; ^∗∗^*p* < 0.01; ^∗∗∗^*p* < 0.001).

## Results

### TfRC Was Accumulated in WDR45-Deficient Cells

**FIGURE 1:**
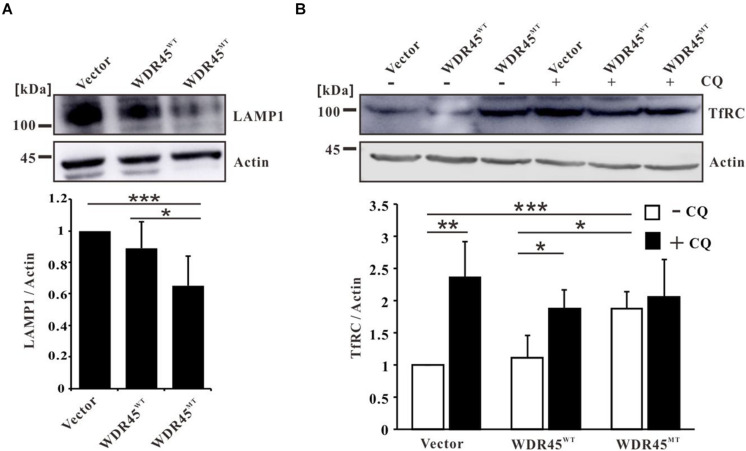
Transferrin receptor (TfRC) was accumulated in WDR45-deficient cells. **(A)** The lysosomal protein LAMP1 was significantly lowered in *WDR45*^*MT*^ expressing HeLa cells. **(B)** TfRC was accumulated in *WDR45*^*MT*^ expressing HeLa cells and in *WDR45*^*WT*^ expressing HeLa cells treatment with 100 μM autophagy inhibitor chloroquine (CQ) for 8 h. *p < 0.05; **p < 0.01; ***p < 0.001.

Earlier, we showed that the overexpression of this mutant *WDR45* in HeLa cells resulted in the accumulation of LC3-II and p62, which suggested an impairment of autophagy (Xiong et al., 2019). Furthermore, consistent with previous studies that lysosome function was impaired in WDR45 mutant fibroblasts (Seibler et al., 2018), we found that the lysosomal protein LAMP1 was significantly lowered in *WDR45*^*MT*^ expressing cells (Figure 1A), suggesting a reduced autophagic degradation rate in the WDR45^*MT*^ cells. By using Western blotting, we confirmed that TfRC was accumulated in the *WDR45*^*MT*^ expressing cells, compared with *WDR45*^*WT*^ expressing and control cells (Figure 1B). Swarup and his colleagues have demonstrated that TfRC could be degraded *via* autophagy (Sirohi et al., 2013, 2015); therefore, we hypothesized that impairment of autophagy by overexpressing *WDR45*^*MT*^ causes the accumulation of TfRC. To confirm this, we investigated the expression of TfRC after treatment with autophagy inhibitor CQ. As we expected, the protein level of TfRC was significantly increased in *WDR45*^*WT*^ expressing and control cells but not in *WDR45*^*MT*^ expressing cells upon treatment with CQ (Figure 1B). Taken together, these results imply that *WDR45* mutation led to the accumulation of TfRC.

### Overexpression of *WDR45*^*MT*^ Impaired the Autophagic Degradation of TfRC

**FIGURE 2:**
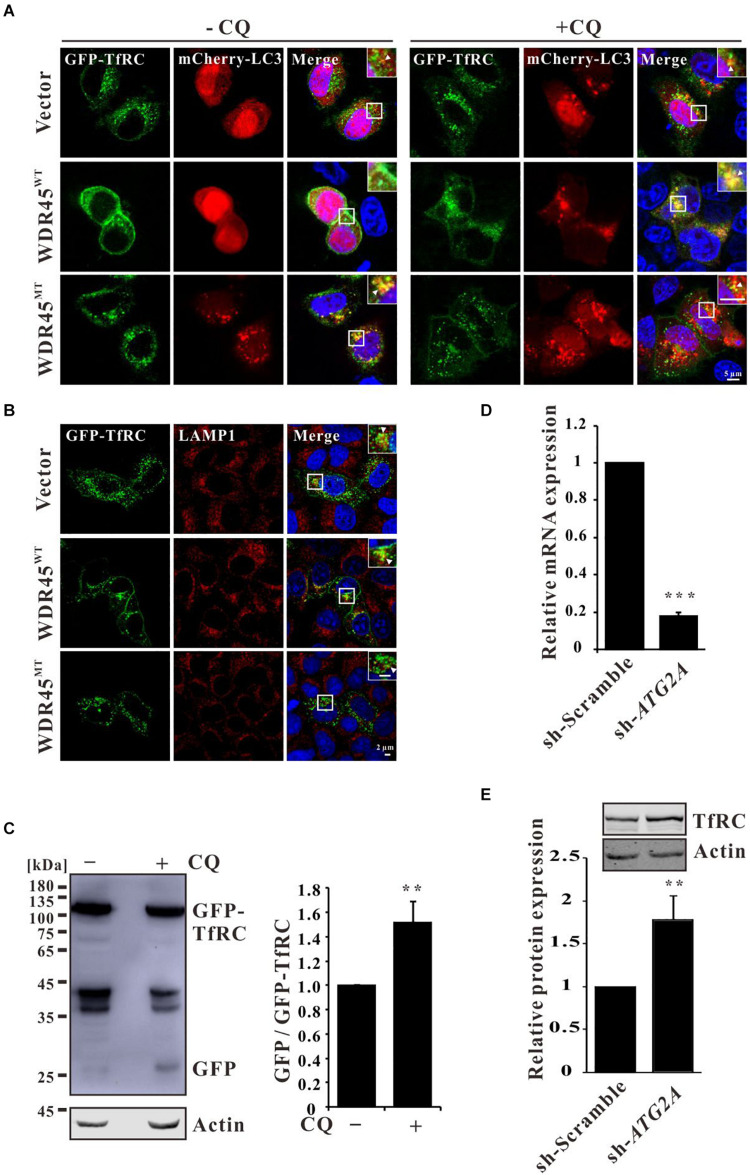
Transferrin receptor (TfRC) was degraded *via* autophagy. **(A)** GFP-TfRC co-localized with autophagosomal marker mCherry-LC3. GFP-TfRC and mCherry-LC3 were coexpressed in HeLa cells and treated with 100 μM chloroquine (CQ) for 8 h, and then the cells were fixed and detected using a confocal microscope. **(B)** GFP-TfRC co-localized with lysosomal marker LAMP1. GFP-TfRC was expressed in HeLa cells, and then the cells were fixed and stained with the anti-LAMP1 antibody. **(C)** Proteolytic cleavage assay was performed to detect GFP moiety. GFP-TfRC was expressed in HEK293T cells and then the cells were treated with 10 μM CQ for 24 h, lysed, and detected by Western blotting using a GFP-specific antibody. **(D)** qPCR analysis of *ATG2A*. **(E)** TfRC was accumulated after the knockdown of *ATG2A*. **p < 0.01 and ***p < 0.001.

To investigate the co-localization of TfRC and autophagy marker LC3, we expressed GFP-TfRC and mCherry-LC3 in HeLa cells which are stably expressed as *WDR45*^*WT*^ or *WDR45*^*MT*^, and the immunofluorescence analysis showed that GFP-TfRC co-localized with mCherry-LC3 in control and *WDR45*^*WT*^ expressing cells after treatment with CQ (Figure 2A). The co-localization of GFP-TfRC and mCherry-LC3 was even observed in the absence of CQ in *WDR45*^*MT*^ expressing cells while the co-localization was rare in control and *WDR45*^*WT*^ expressing cells (Figure 2A). Then, we considered determining whether TfRC is degraded within lysosome. The immunofluorescence results revealed that GFP-TfRC was indeed co-localized with the lysosomal marker LAMP1, suggesting that GFP-TfRC was localized in lysosome (Figure 2B). The autophagic degradation of substrates can be monitored with the GFP cleavage assay (Ni et al., 2011). This assay is based on the observation that the GFP moiety is often cleaved as a whole from GFP-tagged autophagic substrates inside the autolysosome and accumulates because of its relative resistance to further digestion (Xiong et al., 2018). We expressed GFP-TfRC in HEK293T cells and found that upon treatment with CQ the release of free GFP was increased, suggesting that TfRC was degraded within lysosome (Figure 2C). WDR45 has a strong binding capacity for ATG2, thereby recruiting ATG2 to the nascent autophagosome (Maeda et al., 2019). ATG2 bridges membranes and therefore promotes autophagosome biogenesis upon the transfer of lipids from the endoplasmic reticulum (ER) or ATG9 vesicles (Chowdhury et al., 2018; Maeda et al., 2019; Tang et al., 2019; Valverde et al., 2019). Therefore, we inhibited the autophagosome formation by the knockdown of *ATG2A*, and the results showed that TfRC was also increased (Figures 2D,E). Taken together, these results suggest that TfRC was degraded *via* autophagy and *WDR45* mutation impaired the autophagic degradation of TfRC.

### Increased TfRC and Simultaneously Decreased FTH Elevated the Fe^2+^ Level

**FIGURE 3:**
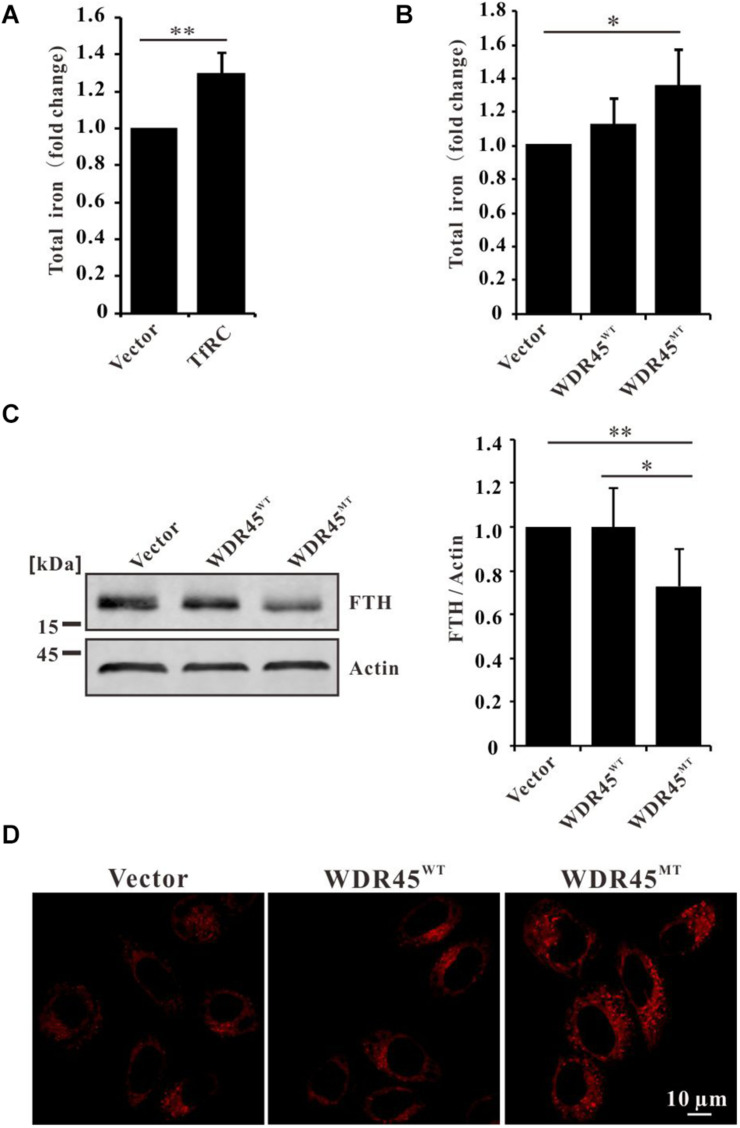
WDR45 deficiency leads to iron overload. **(A)** Total iron content was elevated in HeLa cells overexpression of transferrin receptor (TfRC). **(B)** Total iron content was increased in *WDR45^*MT*^* expressing cells. **(C)** Ferritin H (FTH) chain was significantly lowered in *WDR45*^*MT*^ expressing cells. **(D)** Fe^2+^ was accumulated in *WDR45*^*MT*^ expressing cells. **p < 0.01 and ***p < 0.001.

Overexpression of TfRC increased the cellular iron content (Figure 3A), which is consistent with the previous results (Chirasani et al., 2009; Pereira et al., 2015, 2016). Since TfRC was accumulated in *WDR45*^*MT*^ expressing cells which imply that the cells may uptake more irons into the cells, we examined the total cellular iron content in *WDR45*^*MT*^ stably expressing cells. We observed an increase in the levels of iron content when comparing *WDR45*^*MT*^ expressing cells with control cells (Figure 3B). Ferritin is the major intracellular iron storage protein that consists of two components, namely ferritin L (FTL) chain and FTH chain. Western blot analysis revealed reduced FTH in *WDR45*^*MT*^ expressing cells (Figure 3C). The ferroxidase activity of FTH oxidates redox-active Fe^2+^ to redox-inactive Fe^3+^ (Harrison and Arosio, 1996). The reduced FTH in *WDR45*^*MT*^ expressing cells will lead to increased Fe^2+^. We evaluated the intracellular levels of Fe^2+^ using the specific fluorescent probe FerroOrange under confocal microscopy (Wang and Tang, 2019). As expected, the fluorescent intensity of cells expressing *WDR45*^*MT*^ was significantly increased, confirming that an increase in the concentration of intracellular Fe^2+^ occurs in the *WDR45*^*MT*^ expressing cells (Figure 3D and Supplementary Figure 1). Taken together, these results suggest that WDR45 mutation resulted in increased TfRC but decreased FTH led to Fe^2+^ accumulation.

### *WDR45* Mutation Promoted Ferroptosis

**FIGURE 4:**
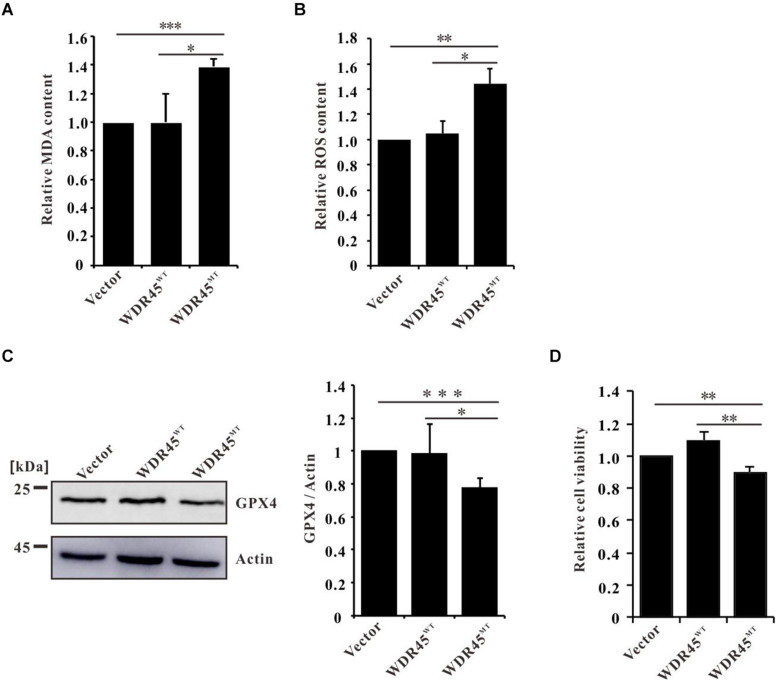
WDR45 deficiency induces ferroptosis. **(A)** Malondialdehyde (MDA) content was significantly increased in *WDR45*^*MT*^ expressing cells. **(B)** Reactive oxygen species (ROS) content was significantly increased in *WDR45*^*MT*^ expressing cells. **(C)** Glutathione peroxidase 4 (GPX4) was significantly lowered in *WDR45*^*MT*^ expressing cells. **(D)** Cell viability was decreased in *WDR45*^*MT*^ expressing cells. *p < 0.05; **p < 0.01; ***p < 0.001.

Ferroptosis is a regulated cell death driven by iron-dependent lipid peroxidation (Dixon et al., 2012). Elevated Fe^2+^ promotes the generation of lipid peroxidation and ROS (Chen et al., 2020). We found that in *WDR45*^*MT*^ expressing cells, the content of MDA (an end product of lipid peroxidation) and ROS was significantly increased (Figures 4A,B). Moreover, we found that GPX4, which is an antioxidant defense enzyme to eliminate toxic lipid hydroperoxides, was also downregulated (Figure 4C). Increased LPO and decreased GPX4 are signatures of ferroptosis (Yang and Stockwell, 2016; Doll and Conrad, 2017). As expected, we detected a significantly reduced cell viability in *WDR45*^*MT*^ expressing cells (Figure 4D). Taken together, these results suggest that *WDR45* mutation promoted ferroptosis.

## Discussion

The patients with BPAN are characterized by global developmental delay in early childhood that is essentially static, with slow motor and cognitive gains until adolescence or early adulthood. In young adulthood, the affected individuals develop progressive dystonia, parkinsonism, extrapyramidal signs, and dementia, resulting in severe disability (Haack et al., 2012; Xiong et al., 2019). In case of the same with other subtypes of neurodegeneration with brain iron accumulation (NBIA), iron accumulation was found in the basal ganglia (Haack et al., 2012). Saitsu et al. (2013) first demonstrated that autophagy deficiency contributes to the pathogenesis of BPAN. The autophagy involves the sequestration of cytoplasm by autophagosome which ultimately fuse with lysosome where their contents are degraded. WDR45 is an ortholog of yeast ATG18 which promotes phagophore membrane expansion by recruiting ATG2 (Bakula et al., 2017, 2018; Chowdhury et al., 2018; Maeda et al., 2019; Tang et al., 2019; Valverde et al., 2019). *WDR45* mutation impairs autophagy by inhibiting the autophagosome formation and reexpression of BPAN-related mutations of *WDR45* fails to rescue the autophagy defects in Wdr45-deficient cells (Ji et al., 2021). Moreover, it showed that *WDR45* mutation also diminished lysosomal function (Seibler et al., 2018) by reducing the protein level of LAMP1 (Figure 1A). These findings suggest that *WDR45* mutation in patients with BPAN resulted in reduced autophagic degradation rate by deceased autophagosome formation and lysosomal degradation ability.

Transferrin receptor is an iron import protein that is essential for most cells (Gammella et al., 2017). It has been previously shown that TfRC could be ubiquitinated and degraded in lysosome (Tachiyama et al., 2011; Fujita et al., 2013). Previous results had revealed that TfRC was degraded *via* autophagy (Sirohi et al., 2013, 2015), while in this study, we showed that TfRC co-localized with the autophagosome and lysosome markers, LC3 and LAMP1 (Figures 2A,B). GFP cleavage assay showed that GFP-TfRC was degraded within lysosome (Figure 2C). These findings confirmed that TfRC was indeed degraded through the autophagy pathway. Therefore, autophagic degradation of TfRC could be blocked by autophagy inhibitor bafilomycin A1 (Horonchik and Wessling-Resnick, 2008; Byrne et al., 2013) and also the lysosomal inhibitor CQ (Figure 1B). Furthermore, TfRC was also accumulated upon the inhibition of autophagosome biogenesis by knockdown of *ATG2A* (Figure 2E). In very recent studies, the results had showed that WDR45 is essential for the autophagosome maturation into autolysosome (Ji et al., 2021), which is consistent with this study results that the co-localization of TfRC and LC3 was observed even in the absence of CQ in *WDR45*^*MT*^ expressing cells (Figure 2A). Taken together, this study results suggested that *WDR45* mutation blocked the autophagic degradation of TfRC, thus leading to the accumulation of TfRC in *WDR45*^*MT*^ expressing cells.

Overexpression of TfRC led to iron overload (Chirasani et al., 2009; Pereira et al., 2015, 2016); therefore, elevated TfRC in *WDR45*^*MT*^ expression cells imported more iron (Figures 3A,B). Chelation of iron by the iron chelator, namely deferoxamine (DFO), induced the autolysosome formation (Rakshit et al., 2020), while iron overload resulted in abnormal autophagosome accumulation and lysosomal loss which impaired autophagy (Jahng et al., 2019; Uberti et al., 2019). Taken together, *WDR45* mutation resulted in iron overload which may, in turn, impair autophagy.

Ferritin is the main iron storage protein that plays a critical role in the regulation of cellular iron metabolism (Harrison and Arosio, 1996). The ferroxidase activity of FTH can prevent Fe^2+^ from partaking in the production of ROS (Winterbourn, 1995). However, we found that FTH was decreased in *WDR45*^*MT*^ overexpression cells (Figure 3C) which is consistent with previous results (Seibler et al., 2018). Nuclear receptor coactivator 4 (NCOA4)-mediated ferritinophagy was found to regulate iron homeostasis, and acceleration of ferritin degradation increased cellular Fe^2+^ (Mancias et al., 2014; Hou et al., 2016). However, patients with BPAN with *WDR45* mutation presented impaired autophagy and lysosomal function (Figure 1A; Saitsu et al., 2013; Seibler et al., 2018; Wan et al., 2019; Xiong et al., 2019), in whom ferritinophagy seems not to be the reason for decreased FTH.

Increased total iron content and reduced FTH consequently resulted in the accumulation of Fe^2+^ (Figure 3D and Supplementary Figure 1) which further promote the generation of LPO and ROS (Figures 4A,B). However, we detected a downregulated GPX4 in *WDR45*^*MT*^ expression cells (Figure 4C). Increased LPO and ROS but decreased GPX4 are signatures of ferroptosis (Yang and Stockwell, 2016; Doll and Conrad, 2017). Inactivation or downregulation of GPX4 results in overwhelming LPO that causes cell ferroptosis (Stockwell et al., 2017). Therefore, cell viability was reduced in *WDR45*^*MT*^ expression cells which suggest that *WDR45* mutation promoted ferroptosis (Figure 4D). Ferroptosis is implicated in the pathological cell death associated with the neurodegeneration diseases such as Alzheimer’s disease (AD), Parkinson’s disease (PD), Huntington’s disease (HD), and amyotrophic lateral sclerosis (ALS; Salvador, 2010; Chen et al., 2015; Do Van et al., 2016; Hirschhorn and Stockwell, 2019; Pena-Bautista et al., 2019). This is the first evidence that *WDR45* mutation promotes ferroptosis which suggests that ferroptosis is involved in BPAN and may contribute to the progression of BPAN.

**FIGURE 5:**
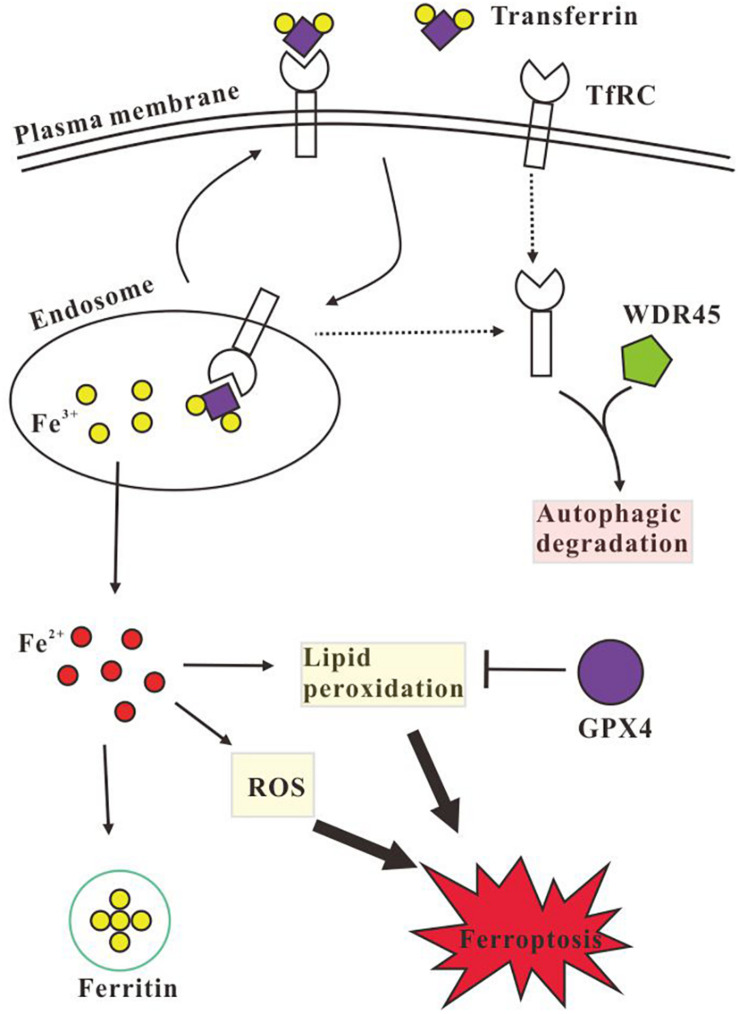
Schematic illustration of the autophagic degradation of transferrin receptor (TfRC) regulates iron homeostasis. TfRC imports iron by binding transferrin, the Fe^3+^ is released inside the endosome, and then Fe^3+^ is transported to the cytoplasm and is reduced to Fe^2+^, while the TfRC is recycled back to the plasma membrane or is degraded *via* autophagy. Excess Fe^2+^ promotes the generation of LPO and reactive oxygen species (ROS) which causes cell ferroptosis. Intracellular excess iron could be stored by ferritin and the LPO could be eliminated by glutathione peroxidase 4 (GPX4). However, WDR45 deficiency impairs autophagic degradation of TfRC and decreases the protein level of ferritin H (FTH) chain and GPX4, therefore the cellular Fe^2+^ is elevated which promotes the generation of LPO and ROS and induces ferroptosis subsequently.

In summary, this study data showed that the mutation (NM_001029896.1, c.1037_1038del) in *WDR45* impaired autophagy and lysosome function; therefore, overexpression of this mutant *WDR45* in HeLa cells resulted in TfRC accumulation and decreased FTH which in turn consequently elevated intracellular iron and further promoted ferroptosis (Figure 5). This study provided a piece of important evidence that the autophagic degradation of TfRC regulates iron homeostasis. These findings will reveal the pathogenesis of brain iron accumulation in patients with BPAN. Further studies are needed to explore whether the induction of autophagy or the inhibition of ferroptosis is a potential strategy for the treatment of BPAN and other NBIAs as well.

## Data Availability Statement

The original contributions presented in the study are included in the article/Supplementary Material, further inquiries can be directed to the corresponding authors.

## Author Contributions

QX, PL, and CW conceptualized the study, designed the experiments, and supervised the study. QX, XL, and WL performed the experiments and wrote the original manuscript. GC and HX analyzed the screen data and revised the manuscript. All authors contributed to the article and approved the submitted version.

## Conflict of Interest

The authors declare that the research was conducted in the absence of any commercial or financial relationships that could be construed as a potential conflict of interest.
