# Supplementary figures and images for: WDR45 Mutation Impairs the Autophagic Degradation of Transferrin Receptor and Promotes Ferroptosis
Source: Front Mol Biosci. 2021 May 3;8:645831. doi: 10.3389/fmolb.2021.645831 (PMC8126626; doi:10.3389/fmolb.2021.645831)

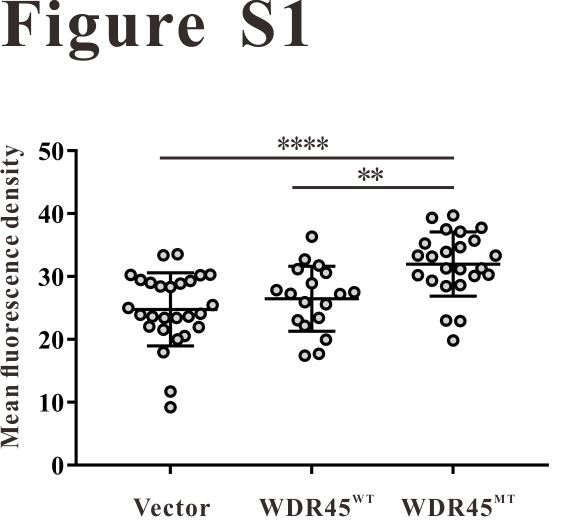

Supplement: Supplementary file 1 [file Image_1.JPEG]
